# Onset of type 1 diabetes during bone growth period is associated with increased prevalence of bone fracture: A post‐hoc analysis of a cross‐sectional study

**DOI:** 10.1111/jdi.13898

**Published:** 2022-09-01

**Authors:** Yuji Komorita, Masae Minami, Yasutaka Maeda, Rie Kishita, Toshiaki Ohkuma, Takanari Kitazono

**Affiliations:** ^1^ Division of General Internal Medicine Kyushu Dental University Kitakyushu Japan; ^2^ Minami Diabetes Clinical Research Center Fukuoka Japan; ^3^ Department of Medicine and Clinical Science, Graduate School of Medical Sciences Kyushu University Fukuoka Japan; ^4^ Clinic Masae Minami Fukuoka Japan

## Abstract

In this single‐center, cross‐sectional study, we demonstrated that the prevalence of fracture was significantly higher in patients who onset type 1 diabetes during 0–4 years, and 10–14 years compared with adult‐onset type 1 diabetes. We are aware that this study contains a lot of limitations including non‐prospective study design and a small number of participants. However, the results of this study, if followed by a larger cohort study, could provide important insights into the increased risk of fracture in patients with type 1 diabetes, and suggest the need for attention and perhaps early intervention for patients with type 1 diabetes who developed during these periods.

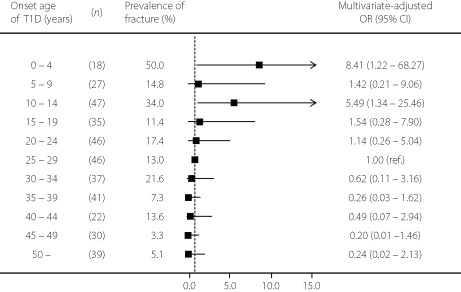

Dear Editor,

Type 1 diabetes is associated with an increased risk of bone fracture, although the full mechanism is to be elucidated. Bone growth is the greatest during two main stages – the infant and pubertal years – when the musculoskeletal system grows the most. Although some recent studies have reported that patients with childhood‐onset type 1 diabetes had greater deficits in bone density^1^, no study has investigated these two stages in detail.

The present single‐center, cross‐sectional study was carried out with 388 Japanese patients aged ≥20 years who had type 1 diabetes and attended the Clinic Masae Minami (Fukuoka, Japan) between October 2019 and April 2020^2^. We evaluated the relationships between onset age of type 1 diabetes and the history of fracture using multivariate‐adjusted logistic regression analysis.

The mean age of participants was 45.1 years, and 42.2% were men. A total of 64 patients experienced fractures after a diagnosis of diabetes. Figure 1 shows the odds ratios for the prevalence of fracture according to the onset age of type 1 diabetes. Compared with those who had onset of type 1 diabetes during 25–29 years, the multivariate‐adjusted odds ratio for fracture was 8.41 (95% confidence interval 1.22–68.27) in patients who had onset of type 1 diabetes aged 0–4 years, and 5.49 (95% confidence interval 1.34–25.46) in patients who had onset of type 1 diabetes aged 10–14 years.

**Figure 1:**
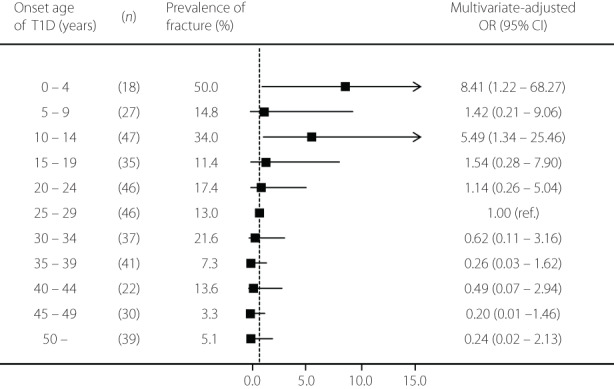
Multivariate‐adjusted odds ratios of the prevalence of fracture according to onset age of type 1 diabetes. Multivariate adjustments include age, sex, body mass index, duration of diabetes, current smoking habit, current drinking habit, exercise habit, family history of hip fracture, hemoglobin A_1c_, diabetic neuropathy, retinopathy, nephropathy and the history of severe hypoglycemia. CI, confidence ratio; OR, odds ratio.

Insulin deficiency and hyperglycemia can affect the bone cells functions, resulting in impairing the bone strength, geometry and microarchitecture^3^. These negative associations are speculated to be greatest just after onset of type 1 diabetes because of the difficulty of optimizing insulin replacement therapy^4^. Thus, developing type 1 diabetes during the periods of greatest bone growth might result in decreased bone formation, and reduced peak bone mass in adulthood. Our study suggests that timing of type 1 diabetes onset might be important for estimating lifetime fracture risk in patients with type 1 diabetes.

We are aware that the present study contains many limitations, including non‐prospective study design and a small number of participants. However, the results of this study, if followed by a larger cohort study, could provide important insights into the increased risk of fracture in patients with type 1 diabetes, and suggest the need for attention and perhaps early intervention for patients who developed type 1 diabetes during these periods.

## DISCLOSURE

The authors declare no conflict of interest.

Approval of the research protocol: The Clinic Masae Minami Institutional Review Board.

Informed consent: Written informed consent was obtained from all the participants.

Approval number: MERC‐19‐001, Approval date: 1 October 2019.

Animal studies: N/A.
